# Therapeutic Efficacy of Multi-Characteristic Opsin Gene Therapy in a Mouse Model of Stargardt Disease

**DOI:** 10.3390/bioengineering13060660

**Published:** 2026-06-04

**Authors:** Samarendra Mohanty, Subrata Batabyal, Sanghoon Kim, Michael Carlson, Adnan Dibas

**Affiliations:** 1 Nanoscope Therapeutics Inc., 2777 N. Stemmons Fwy, Dallas, TX 75207, USA; 2 Nanoscope Technologies LLC., 1312 Brown Trail, Bedford, TX 76022, USA

**Keywords:** multi-characteristic opsin, stargardt disease, gene therapy, macular degeneration

## Abstract

Optogenetic gene therapy-based treatment offers a unique approach to bypass dysfunctional or degenerated photoreceptors in retinal degenerative disorders. Ambient light-activatable multi-characteristic opsin (MCO) targeted to bipolar cells of the retina has demonstrated partial vision restoration in animal models of retinitis pigmentosa (RP). Here, we describe the potential therapeutic efficacy of intravitreally delivered AAV-carried MCO-010 in a mouse model of Stargardt disease. MCO-010 treatment led to significantly improved behavioral outcomes in the visually guided radial arm water maze. Furthermore, longitudinal optical coherence tomographic imaging showed that the MCO-010 treatment led to no notable change in the retina thickness. Furthermore, the MCO-010-treated mice exhibited higher electrophysiological responses compared to the control group. Together, these findings demonstrate potential vision-restoring and disease-modifying aspects of ambient light-activatable intravitreal MCO-010 therapy.

## 1. Introduction

Stargardt disease (STGD1), an inherited form of juvenile macular degeneration, affects approximately 1 in 8000–10,000 people [1]. This genetic disorder, which is the most common form of inherited juvenile macular degeneration, typically manifests in childhood or young adulthood, leading to progressive central vision loss. Mutations in the Adenosine triphosphate (ATP) Binding Cassette Subfamily A Member 4 (*ABCA4*) gene disrupt the function of an essential membrane transporter protein, leading to pathological accumulation of lipofuscin in the macula [2,3,4,5]. This accumulation triggers oxidative stress and progressive damage, resulting in bilateral degeneration of multiple retinal layers. The deterioration particularly affects the outer retina, including photoreceptors, retinal pigment epithelium (RPE), and the underlying choroidal vasculature, ultimately compromising central vision [6,7]. Stargardt disease poses severe vision loss for affected individuals even in adolescence, and there is no approved treatment to date. The treatment of Stargardt disease through conventional gene replacement therapy faces a significant challenge due to the large size of the *ABCA4* gene exceeding the packaging capacity of standard viral vectors. While adeno-associated virus (AAV) vectors are the preferred choice for retinal gene therapy due to their safety profile and tropism, their limited payload capacity (~4.7 kb) cannot accommodate the full *ABCA4* gene sequence, making this approach technically unfeasible for treating Stargardt disease through direct gene replacement [8]. Therefore, alternative therapeutic approaches include delivery of truncated *ABCA4* genes, dual-vector systems for intracellular splicing, or non-viral delivery methods to overcome the size limitation [9,10]. Though lentiviral vectors have higher payload capacity, the clinical trial (NCT01367444) employing lentivirus for subretinal delivery of *ABCA4* cDNA has not been successful [10]. More recently, alternative strategies such as dual-vector and intein-based approaches [11], including programs currently under clinical evaluation for *ABCA4*-associated Stargardt disease, have been developed to address the packaging limitations of the large *ABCA4* gene. Genome editing tools, including Clustered Regularly Interspaced Short Palindromic Repeats (CRISPR) [12], have enabled the correction of single mutation(s); however, multiple genetic mutations across the large *ABCA4* gene are challenging to be corrected by these genome editing tools [13]. STGD1 and dry age-related macular degeneration (dry AMD) have significant overlap in the genetic perturbations in *ABCA4*, molecular, and clinical phenotypes, including accumulation of lipofuscin in the macula, and RPE cell damage [14].

The design of MCO-010 reflects a broader effort to engineer biologically optimized therapeutic systems that integrate targeted delivery, cell-specific expression, and functional restoration. MCO-010 is a multi-characteristic opsin optimized for broadband and fast ambient-light responsiveness, wherein the expression of opsin is driven by the mGluR6 promoter-enhancer system to selectively target retinal ON-bipolar cells following intravitreal AAV2 delivery. This approach was designed to improve pan-retinal expression efficiency and therapeutic function while minimizing the need for surgical (subretinal) [15,16].

By delivering ambient light-sensitive multi-characteristic opsin (MCO) encoding genes using adeno-associated virus-2 (AAV2) to the surviving higher-order retinal neurons, partial restoration of vision in animal models of retinitis pigmentosa (RP) has been reported [17]. The proximity of bipolar cells to the photoreceptor layer in the healthy retina, as well as bipolar cells’ relative number and distribution, provides the potential for greater visual acuity compared to targeting retinal ganglion cells further downstream. MCO-010 uses a viral vector and promoter-enhancer to target bipolar cells, thereby allowing these cells to be light-sensitive in advanced retinal degenerations with permanent photoreceptor loss. The bipolar cells expressing MCO-010 thus gain the ability to function as de facto photoreceptors, which initiate the visual transduction and transmission of the signal through the remaining retinal layers in the photoreceptor-degenerated retina and optic nerve to the brain. Unlike classical gene-specific therapies, the MCO-010 therapy is agnostic to any underlying gene mutation and does not require the presence of outer retinal cells such as rods, cones, or retinal pigment epithelium. By targeting surviving inner retinal neurons rather than correcting a specific genetic defect in deteriorating outer retina, MCO-010 may have broader applicability across multiple retinal degenerative diseases beyond ABCA4-associated Stargardt disease. This mutation-independent approach may be particularly relevant for disorders such as geographic atrophy affecting the macula, in which inner retinal circuitry remains relatively preserved, supporting the potential utility of optogenetic therapies as a broadly applicable platform for restoring visual function across diverse outer retinal degenerative conditions. The bipolar cells are known to remain largely functional despite the disorganization of the retinal architecture that occurs over decades of disease progression. The randomized clinical trial on intravitreally injected MCO-010 in RP has shown the translational success of this approach, with a durable response without eliciting any safety concerns [18]. The current study investigated the ability of the intravitreal injection of AAV2-packaged MCO (MCO-010) to enhance visual responses in the *Abca4^-/-^* mouse strain without any deterioration in retinal structure.

## 2. Methods

All experimental procedures were conducted following the Nanoscope Technologies-Institutional Animal Care and Use Committee-approved protocol. To avoid bias, the imaging, behavioral assays, and electrophysiological measurements, as well as analysis, were performed by individuals masked to the treatments.

### 2.1. Animals

The *Abca4* (*Abca4^tm1Ght^*/J) mice were obtained from Jackson Laboratories [19] and bred and maintained in the animal facility at Nanoscope Technologies. The animals were treated humanely in strict compliance with the Institutional Animal Care and Use Committee (IACUC) on the use of animals in research. The mice were kept under the care of full-time animal care staff at Nanoscope Animal Facility (OLAW assured). The animals are monitored for general health at least once daily by the animal care staff. Mice were maintained on a 12 h:12 h light:dark cycle.

### 2.2. Enhancing Visual Responses by Optogenetics

**Figure 1:**
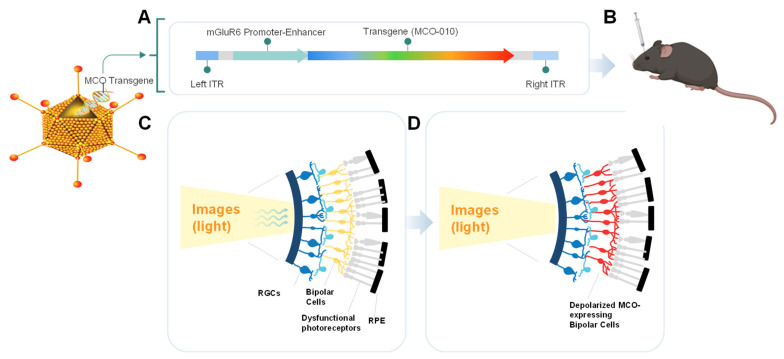
**Principle of enhancing visual responses by optogenetics in Stargardt disease.** (**A**) Multi-Characteristic Opsin (MCO) gene, driven by mGluR6 promoter-enhancer and packaged in AAV2, is delivered intravitreally (**B**). (**C**) Transduced ON-bipolar cells are (**D**) depolarized at broadband ambient light levels, leading to transduction of the visual signal through ganglion cells to the brain via the optic nerve. RGC: Retinal Ganglion Cell, RPE: Retinal Pigment Epithelium.

Figure 1 shows the principle of enhancing visual responses by optogenetics in Stargardt disease. The gene encoding the ambient light activatable Multi-Characteristic Opsin (MCO), driven by mGluR6 promoter-enhancer and packaged in AAV2 (Figure 1A), is delivered intravitreally (Figure 1B). The transduced ON-bipolar cells (Figure 1C) are depolarized at a broadband ambient light level (Figure 1D), leading to transduction of the visual signal through ganglion cells to the brain via the optic nerve.

### 2.3. Screening via Water Maze and Randomization to Groups

*Abca4^-/-^* mice (4 months old) were trained for 2 weeks in a radial arm water maze before establishing the baseline. Briefly, mice were placed into the center of the maze, and a platform was placed just above the water’s surface at the end of one of the arms. The mice rapidly learned to determine the location of the platform by utilizing visual cues (LEDs emitting light in the visible spectrum). The platform (in one of the arms) provided a reward to them where they could rest instead of having to swim. The time to reach the platform was quantified in the presence of a visual-guidance cue. To avoid the inclusion of *Abca4* mice that were able to find the platform at <20 s, the mice were screened through a radial water maze. The exclusion criterion consists of a mouse that does not swim (and rather floats). In total, 14 out of 18 screened mice were selected for the experiment and split into two groups. After the randomized allocation of animals to the treatments (7 for the MCO-010 injection and 7 for the Control-AAV2 injection), animals, samples, and treatments were coded until the data were analyzed.

### 2.4. Randomization and Masking

A randomized block experimental design was used to ensure that the animals had an equal and known probability of being assigned to each treatment group, i.e., MCO-010 or AAV-Control. Animals were allocated to treatment groups using restricted (block) randomization prior to injection. Group assignment, cage number, and mouse identification numbers were determined before the start of the experiment. Animal cages were placed on the shelves in a randomized order, and injections were administered in a random sequence. To minimize bias, personnel conducting the water maze, ERG, and analyses involving retinal thickness measurements were blinded to treatment allocation. Following randomization, animals, samples, and treatments were coded until data analysis was complete.

### 2.5. Intravitreal Injection

A digital weighing scale was used to measure the body weight of the mouse, and depending on the body weight, a ketamine cocktail (K/X/A) was administered intraperitoneally (2.5–3 μL/gm body weight). The animals were anesthetized with a mixture of ketamine (65 mg/kg), xylazine (7.5 mg/kg), and acepromazine (0.5 mg/kg) or isoflurane (2–3%).

Aseptic techniques were used for all surgical procedures, and surgical tools were sterilized in an autoclave. The mice were anesthetized via isoflurane, and local anesthesia was instilled into the eye of the animal. One drop each of 0.5% Proparacaine ophthalmic solution was instilled into the eye of the animal prior to the intraocular injection. The MCO-010 (1.7 × 10^9^ vg/eye) or AAV2-control (1.7 × 10^9^ vg/eye) solution was injected by a sterilized needle (a Hamilton micro-syringe with a 33-gauge needle) inserted through the sclera into the vitreous cavity for the intravitreal injection. Ciprofloxacin 0.3% ophthalmic solution (an antibiotic) was applied after injection to reduce the risk of infection.

### 2.6. Water Maze Behavioral Assessment

The mouse rapidly learns to determine the location of the platform by utilizing visual cues (LEDs, emitting white light) in the radial arm water maze. For each mouse, three trials were conducted in each location (i.e., center, side, and arm of the water maze). The selection of the dropping site (center, side, arm) was random for each trial. Data (video) recording was stopped once the mice found the platform or after 60 s of placing the mice in water to prevent fatigue-induced drowning. Following baseline recording, water maze behavioral recordings were conducted every 4 weeks after intravitreal injection of MCO-010 or AAV2-control. The latency was measured longitudinally for different placement locations.

### 2.7. Optical Coherence Tomography (OCT) Imaging and Analysis of Retinal Thickness

Longitudinal optical imaging using spectral-domain OCT (SDOCT) was carried out to monitor any changes in ocular structure due to MCO-010 treatment as compared to vehicle control. For performing the OCT imaging, mice were anesthetized with a mixture of ketamine (65 mg/kg), xylazine (7.5 mg/kg), and acepromazine (0.5 mg/kg) and mounted on a maneuverable imaging platform. Briefly, 1–2 drops of 1% tropicamide were topically applied to the eyes for pupil dilation. The cornea was kept moist with a balanced salt solution during the measurement period. SD-OCT imaging was performed using NS-NEEL (Nanoscope Instruments Inc., Bedford, TX, USA). B-scan images of the retina at baseline and after intravitreal MCO-010 injection in *Abca4* mice were collected. Retinal thickness measurements were made at different locations of the retina before and after MCO-010 injection. NIH ImageJ software Version 1.54t was used to analyze the B-scan images.

OCT B-scan images were analyzed using ImageJ software (NIH) to quantify retinal thickness (for inner and outer retinal layers). Measurements were taken at multiple locations (keeping the locations for different groups the same to avoid any bias) across the central retina. On average, thirty (30) B-scan images were captured per experiment. The B-scan images with the optic nerve head located at the center of the scan were analyzed. The optic nerve head region in the B-scan was excluded from retinal thickness measurements due to its anatomically unstructured morphology, which lacks the organized laminar organization of the retina and would not yield reliable thickness measurements. Multiple measurements were averaged per image to ensure reproducibility. In addition, multiple analysts measured the retinal thickness using ImageJ, and the Bland–Altman analysis was performed to confirm the agreement between analysts. The mean thickness of the inner and outer retina was calculated by averaging five (5) multi-point measures.

### 2.8. Assessment of Electroretinogram (ERG) Response

After overnight dark adaptation of the *Abca4^-/-^* mice, the mice were anesthetized with Isoflurane, and the pupil was dilated using tropicamide. A heating pad was used at 35 °C, and the mouse was placed on the heating pad after being anesthetized. The cornea was kept moist with a balanced salt solution during the measuring procedure. A ground needle electrode was placed on the base of the tail, with the electrode needle sub-dermally placed between the eyes.

ERG recordings were performed under scotopic conditions using a commercial ERG acquisition system (NS-NEEL, Nanoscope Instruments) using light intensities of 0.01, 0.1, 3, 6, and 25 cd. s/m^2^. For each animal and each flash intensity, 10 individual ERG traces were recorded (subjected to a 60 Hz notch filter, and a 0.1–300 Hz bandpass filter) and averaged to improve the signal-to-noise ratio. No normalization was performed on the raw data. The average waveform was used for all subsequent analyses. The a-wave amplitude was calculated as the difference between the baseline voltage (measured immediately prior to flash onset) and the most negative deflection following the light stimulus. The b-wave amplitude was calculated as the voltage difference between the a-wave trough and the subsequent maximal positive peak of the averaged waveform. Waveform analysis was performed using standard ERG analysis tools provided with the NS-NEEL acquisition software. Longitudinal comparisons of b-wave amplitudes were performed across time points and treatment groups using averaged values for each animal.

### 2.9. Immunohistochemistry

The eyes were fixed in modified Davidson solution overnight and finally stored in 1xPBS. The retinal sections were prepared using a microtome. Some histological sections were processed with H&E staining. Other retinal sections were subjected to 0.5% Triton X-100 (washing solution) three times. The nonspecific binding of antibodies was blocked by 4% serum for 60 min and washed with washing solution three times. The samples were incubated with primary antibodies (1:500 dilution), e.g., anti-mCherry (NBP1-96752; Novus Biologicals, Centennial, CO, USA), PKCα (PA5-17551; ThermoFisher Scientific, Waltham, MA, USA), overnight at 4 °C. After washing the samples with 0.5% Triton X-100 solution in 1xPBS three times, secondary antibody (Goat anti-rabbit Alexa 488 nm, ThermoFisher: A-11008, Goat anti-mouse Alexa 594 nm, ThermoFisher: A-11005), diluted 1:250 in washing solution, was loaded for one hour at room temperature. The samples were stained with nuclei stain DAPI (1:200 dilution) and imaged using fluorescence microscopy.

### 2.10. Fluorescence Analysis

For evaluation of MCO-010 expression after the end-of-study period (24 weeks post-injection), visualization of the reporter (mCherry, which is an integral part of MCO-010) fluorescence was carried out by confocal fluorescence imaging (Olympus, Tokyo, Japan, FluoView 1000) of the immunostained retina slices. Using ImageJ (NIH), four circular regions of interest (ROI) of the same size were selected in the inner nuclear layer in the images, and the average fluorescence intensity was calculated for each image.

## 3. Results

**Figure 2:**
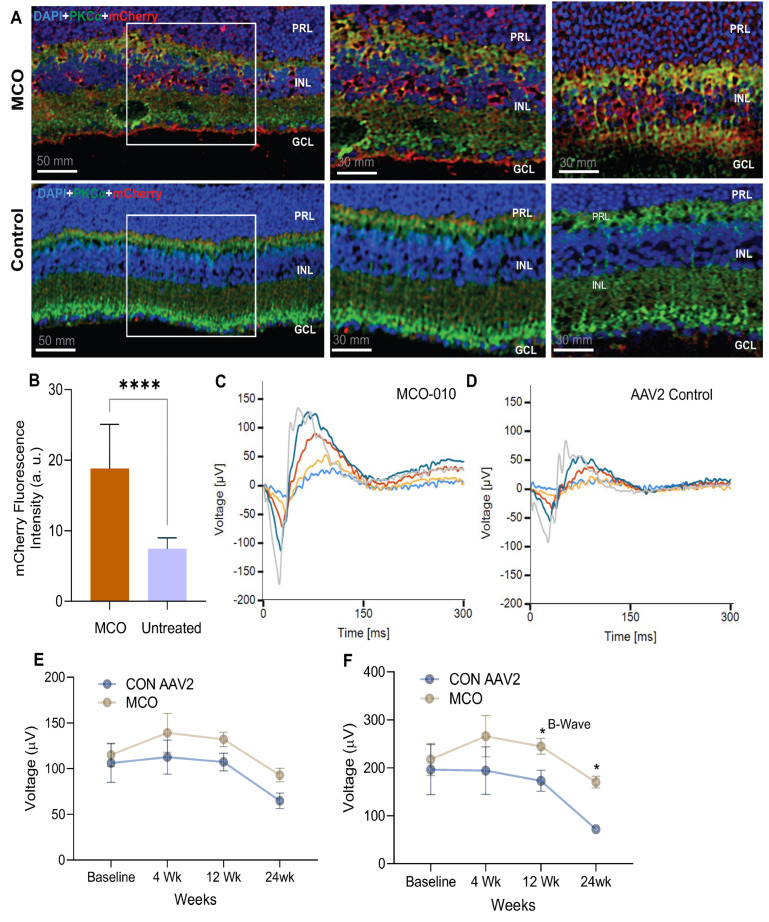
MCO-010 intravitreal injection led to robust expression and enhanced electroretinogram in *Abca4* mice, in contrast to vehicle control. (**A**) Immunostained images of MCO-010-treated and control mice. Expression of MCO-010 in the inner retina of *Abca4* mouse retina is visualized by MCO-010-reporter mCherry. Bipolar cells were stained by PKCα (shown in green), and the mCherry was stained using anti-mCherry antibody (shown in red). Nuclear stain DAPI is in blue. PRL: Photoreceptor layer; INL: Inner nuclear layer; GCL: Ganglion cell layer. Images taken at the end of the study (24 weeks after injection), age of mouse: 42 weeks. (**B**) Quantification of MCO-010 reporter (mCherry) expression in the MCO-010 injected mice group compared with the non-specific red fluorescence of AAV2 vehicle control, N = 4/group, Average ± SEM. Significantly higher reporter fluorescence (**** *p* < 0.0001) was observed in the MCO-010 injected group as compared to the untreated group. Representative scotopic ERG profiles (average of 10 sweeps) in response to white light stimulation at 0.01 (blue line), 0.1 (yellow line), 3 (red line), 6 (teal line), and 25 (gray line) cd. s/m^2^ in (**C**) MCO-010 treated and (**D**) AAV2 vehicle control, *Abca4* mice at 12 weeks after injection (age: 30 weeks). Longitudinally measured (**E**) a-wave, and (**F**) b-wave amplitude of AAV2 vehicle control and MCO-010 treated *Abca4* mice (for 25 cd. s/m^2^). Baseline age: 18 weeks. N = 4/group, Average ± SEM, * *p* < 0.05.

The expression of MCO-010 was quantified using fluorescence intensity of the reporter (mCherry) in the inner nuclear layer (marked by PKCα positive region) of the immunostained retina sections of *Abca4* mouse (Figure 2A). Figure 2B shows the quantification of MCO-010 reporter (mCherry) expression in the MCO-010 injected mice group compared with the non-specific red fluorescence in the AAV2 vehicle control group. As expected, significantly higher reporter fluorescence was observed in the MCO-010-injected group as compared to the control group. To evaluate the impact of MCO-010 treatment on the functioning of the retina, electroretinograms in *Abca4* mice were longitudinally measured in MCO-010-treated and control mice. Raw electroretinogram profiles from *Abca4* mice intravitreally injected with vehicle control or MCO-010 are shown in Supplementary Figure S1. Representative scotopic ERG profiles in response to white light stimulation at 0.01, 0.1, 3, 6, and 25 cd. s/m^2^ in MCO-010-treated mice are shown in Figure 2C. Figure 2D shows the scotopic ERG profiles of AAV2 vehicle control *Abca4* mice in response to various white light stimulation intensities at 12 weeks after injection. Figure 2E shows the quantification of the longitudinally measured b-wave amplitude of AAV2 vehicle control and MCO-010-treated *Abca4* mice. A statistically significant difference in b-wave amplitude was observed at and after 12 weeks between the two groups. These results show that MCO-010 intravitreal injection leads to better-preserved electroretinogram in *Abca4* mice as compared to vehicle control.

**Figure 3:**
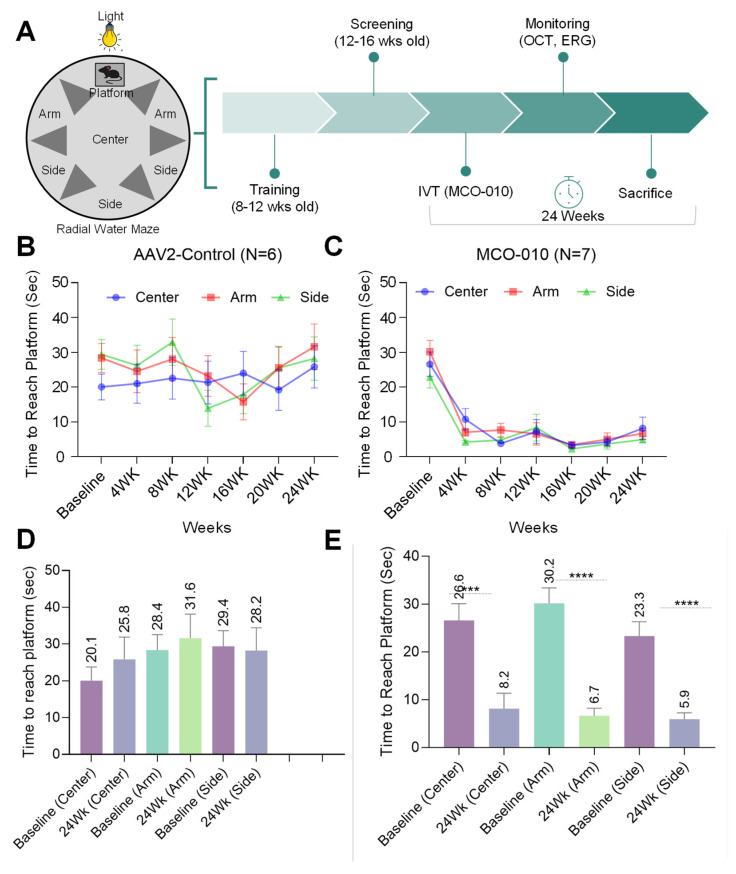
**Intravitreal injection of MCO-010 in *****Abca4***** mice led to significant improvement in ambient-light guided locomotion.** (**A**) Schematic of radial water-maze setup. Time to reach the platform (latency) by the *Abca4* mice from the Center (light intensity: 7 µW/mm^2^), Side, and Arm (5 µW/mm^2^) of the water maze: (**B**) AAV2-control (N = 6), and (**C**) 1.7 × 10^9^ gc/eye MCO-010 (1.14 × 10^12^ vg/mL, 1.5 µL) injected mice (N = 7). Quantitative comparison of latency (from Center, Side, and Arm) at baseline (before injection) and 24 Weeks after injection (age: 42 weeks) in AAV2-control (**D**), and MCO-010 (**E**) treated *Abca4* mice. Av. ± SEM, *** *p *< 0.0005, **** *p* < 0.0001.

To evaluate the therapeutic efficacy of MCO-010 in enhancing visual responses in *Abca4* mice, the latency to find the lighted platform via visually guided spatial navigation in a radial water maze was quantified. A schematic of the radial water-maze setup and timeline is shown in Figure 3A. Longitudinal measurements showed that there was no change in latency to find the platform by the mice injected with AAV2-control, implying that their performance in the light-guided behavior remained the same irrespective of the starting position with respect to the lighted platform (Figure 3B). Supplementary Figure S2 shows Time-lapse pictures of an *Abca4* mouse in the radial arm water maze before and after AAV2-Vehicle injection. As shown in Supplementary Figure S3, there was no change in the latency of the noninjected control *Abca4* mice to find the lighted platform in the radial water maze, providing evidence of consistency of behaviors over repeated measures. The time-lapse pictures of an *Abca4* mouse in a radial arm water maze before and after MCO-010 injection are shown in Supplementary Figure S4. As shown, at 4 weeks after MCO-010 injection, the *Abca4* mice took less time to reach the lighted platform as compared to baseline, not only from the center but from the side arms (Figure 3C). These improvements were observed in light levels similar to ambient light levels and lasted during the whole duration (24 weeks) of the study. Figure 3D shows a quantitative comparison of latency from different starting positions (Center, Side, and Arm) with no significant improvement in AAV2-control mice (N = 6). MCO-010-treated *Abca4* mice (N = 7) exhibited statistically significant differences at Week 24 compared to baseline (Figure 3E).

**Figure 4:**
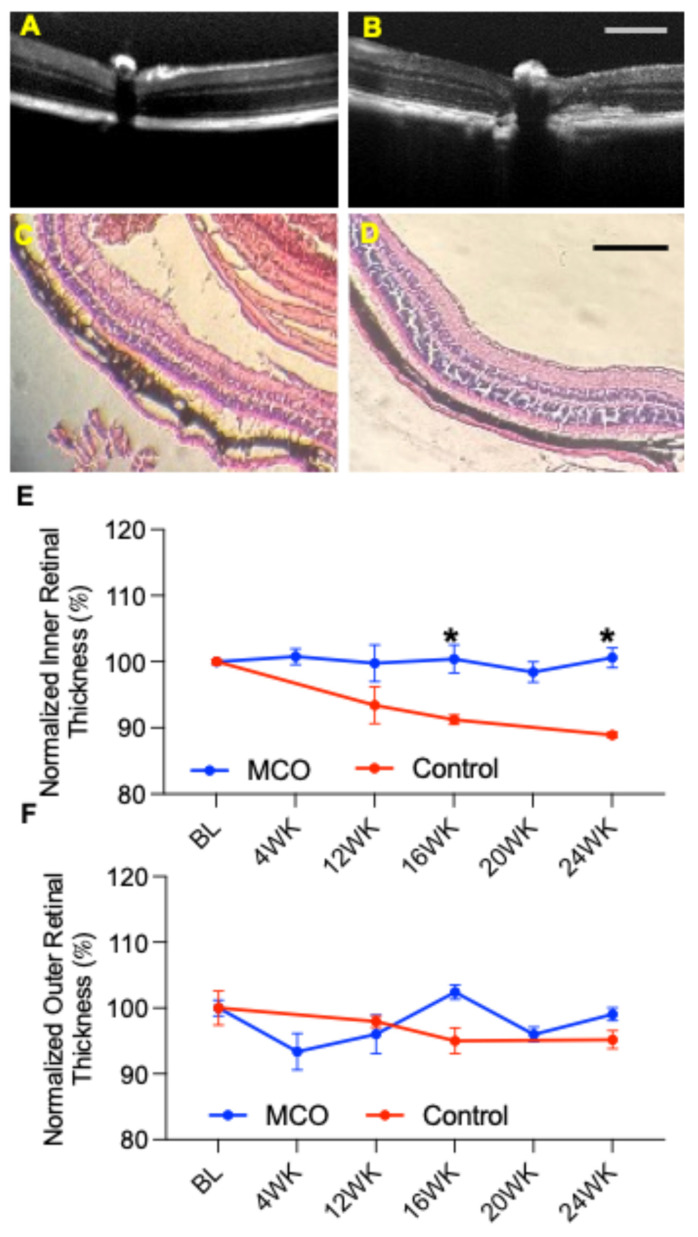
**Longitudinal monitoring of the retina of *****Abca4***** mice with SDOCT with and without MCO-010 injection.** OCT B-scan image at (**A**) baseline and (**B**) after 1.7 × 10^9^ gc/eye MCO-010 injection. Baseline (BL) age: 18 weeks. Scale bar: 300 µm. H&E-stained images of histological sections showing the retina of (**C**) MCO-010-treated and (**D**) Control mice. Scale bar: 300 µm. (**E**) Longitudinally measured inner retina thickness at baseline and post-injection time points (normalized to baseline). N = 4/group, Average ± SEM, * *p* < 0.05. No notable change in retina thickness in the MCO-010-injected eye compared to the control group. (**F**) Longitudinally measured outer retina thickness at baseline and post-injection time points. N = 4/group, Average ± SEM.

Since attenuation of degeneration has been observed in animal models of retinitis pigmentosa upon intravitreal injection of MCO-010, monitoring of retinal thickness was conducted in *Abca4* mice. Figure 4A shows an OCT B-scan image of 4-month-old *Abca4* mice at baseline. As shown in Figure 4B and Supplementary Figure S5, the retina structure remained intact at 24 weeks after MCO-010 injection for the 10-month-old *Abca4* mice. Figure 4C and Figure 4D show the H&E-stained retina for MCO-010 and control mice, respectively. Longitudinal monitoring of the retina of control *Abca4* mice without MCO-010 injection showed a decline of inner retina thickness at and after 8 months of age (Figure 4E, Supplementary Figure S5). In contrast, the longitudinally measured retinal thickness at baseline and post-injection time points in MCO-010-treated mice shows no notable change in retina thickness in the MCO-010-injected eye as compared to the control group. There was no significant decline in outer retina thickness observed in either of the groups (Figure 4F).

## 4. Discussion

Mutations in ATP Binding Cassette Subfamily A Member 4 (ABCA4) are associated with late-onset dry age-related macular degeneration (dry-AMD) and early-onset Stargardt Disease (STGD1). *Abca4^-/-^* animal models are crucial for studying STGD1, which is caused by mutations in the *Abca4* gene [14,20]. *Abca4^tm1Ght^*/J mice used in this study have a neo cassette replacing the promoter and exon 1 of the ATP-binding cassette, sub-family A (ABC1), member 4 (*ABCA4*) gene, abolishing gene expression. ABCA4 (ABCR) is a retina-specific protein localized in the outer segment disk edges of rod photoreceptors [21]. Mutations in *ABCA4* have been linked to the onset of Stargardt macular dystrophy [22]. ABCA4 acts as a transmembrane flippase transporter for phosphatidylethanolamine (N-Ret-PE), which moves N-Ret-PE from inside the photoreceptor disks out to the cytoplasmic surface [23]. The major bis-retinoid-lipofuscin pigment in the retinal pigment epithelium (RPE) of *Abca4^-/-^* mice and STGD patients is A2E [10]. The knockout mice exhibit slow-photoreceptor degeneration and delayed dark adaptation following a photobleach [24,25]. Besides natural aging, since the mice were maintained on a 12 h:12 h light: dark cycle, the prolonged light exposure could have caused additional stress for the observed retinal degeneration.

Instead of replacing or correcting the large *ABCA4* gene in the photoreceptors, we photosensitized the retinal bipolar cells with synthetic MCO-010 that led to no change in retinal thickness, while improving the visual function. In the visual pathway, signals generated by the photoreceptors are transmitted to bipolar cells, which then relay information to the brain via RGCs and the optic nerve. In MCO-transduced *Abca4^-/-^* mice, MCO-expressing bipolar cells act as de facto photoreceptors that directly respond to light and transmit signals through the RGCs. Behavioral vision tests showing improved functional vision in MCO-treated mice indicate that the higher-order visual circuitry can process the signal generated by the reengineered retina for vision sensation. Also, our previous study on MCO-010 has shown improvement in visual-evoked potentials (VEP) in the visual cortex after MCO-010 treatment [26], demonstrating that signals originate in bipolar cells and successfully propagate through RGCs to reach central visual areas. Since the improved function of retina was observed in MCO-010-treated animals, the ambient light activation of MCO-010-expressing ON-bipolar cells is believed to overcome the self-generated oscillations in the cone ON-bipolar cells of the degenerating retina that could potentially deteriorate the visual signal [27].

Use of a proprietary RepCap plasmid and mGluR6 bipolar-cell-targeted promoter-enhancer enabled robust transduction of the inner retina upon intravitreal MCO-010 delivery [17,26,28]. In the *Abca4^-/-^* mouse model used in this study, MCO-010 expression was primarily observed in ON-bipolar cells, consistent with the targeting specificity of the mGluR6 promoter-enhancer system. The MCO-010 therapeutic strategy is designed to selectively sensitize surviving bipolar cells rather than photoreceptors, thereby enabling restoration of visual signaling through preserved inner retinal circuitry. These findings support the mutation-independent and bipolar-cell-targeted mechanism underlying MCO-010-mediated optogenetic therapy. Longitudinal monitoring of retinal thickness by OCT revealed no change in retinal thickness following MCO-010 injection. This effect was primarily observed in the inner retina, implying prevention of further disorganization of retinal layer(s). Since remodeling in the photoreceptor-dysfunctioning retina is known to occur due to deafferentiation [29], MCO-010 sensitization of bipolar cells, leading to bipolar cell activities, may be able to mitigate maladaptive remodeling, which is manifested in the maintenance of the inner retinal thickness. Although MCO-010 does not replace lost photoreceptors, preservation of remaining retinal architecture together with restoration of visual function suggests the potential stabilization of retinal circuitry and mitigation of remodeling associated with progressive outer retinal degeneration, supporting a possible disease-modifying effect beyond symptomatic functional compensation. This is similar to that observed by us in fast-degenerating animal models of retinitis pigmentosa (RP) treated with MCO-010 [26]. Since MCO-010 is targeted to bipolar cells, the results reported here confirm the previously observed effect and, therefore, hold the potential to slow degeneration in all outer retinal degenerative diseases [30]. Protection of retinal degeneration upon optogenetic sensitization of retina has also been reported by Katada et al. [31]. This disease-modifying aspect is yet to be confirmed in the MCO-010 clinical trials on RP and Stargardt disease. However, this assessment would require significant long-term follow-up of the slowly degenerating patients treated with MCO-010, along with a natural history of the matched control subjects.

The development of MCO-010 reflects a broader trend toward bioengineered therapeutic platforms that integrate targeted delivery, cell-specific expression, and functional restoration. In this study, the expression of a multi-characteristic opsin optimized for broadband ambient-light activation was driven by the mGluR6 promoter-enhancer system to selectively sensitize retinal ON-bipolar cells following intravitreal AAV2 delivery.

Since AAV vectors have limited packaging capacity, efficient transgene design and expression optimization remain critical considerations for next-generation retinal gene therapies. In the case of MCO-010, the multi-characteristic opsin design required optimization of the promoter-enhancer system and vector configuration to achieve large transgene packaging and delivery, achieving therapeutically relevant expression while maintaining compatibility with intravitreal delivery of a single AAV construct. Recent advances in engineered biological systems have emphasized the importance of improving expression efficiency, targeting specificity, functional output, and long-term therapeutic stability to enhance overall therapeutic efficacy. For example, improved recombinant protein expression and performance in microbial systems have been achieved through engineering of biological machinery [15] and such engineered therapeutic platforms have demonstrated durable and sustained therapeutic activity through optimized cellular targeting and functional stability [32].

The functional improvement, measured objectively by scotopic ERG and light-guided behavior in *Abca4^-/-^* mice, was evident in the MCO-010-dosed animals as compared to the control. Collectively, vision improvement was observed in *Abca4^-/-^* mice model of degeneration, even at an advanced stage (10 months old, equivalent to several decades of human life). These improvements were observed at ambient light levels, owing to the unique characteristics of multi-characteristic opsin (MCO) being highly sensitive to broadband light with fast kinetics [17,28]. The functional improvements observed with MCO-010 in the *Abca4^-/-^* model should be interpreted in the context of the model’s characteristics. Unlike severe degenerative models with photoreceptor loss, *Abca4^-/-^* mice maintain residual photoreceptor function. Therefore, the enhanced visual responses observed following MCO-010 treatment represent an additive effect, where MCO-sensitized bipolar cells complement the remaining natural photoreceptor-mediated vision. We have previously demonstrated the utilization of this novel chimeric MCO-010 protein for restoring vision in fast-degenerating *rd1* and slow-degenerating *rd10* animal models. The safety and toxicity of intravitreally delivered MCO-010 have also been established in wild-type dogs [33,34].

Recent advances in engineered therapeutic platforms have increasingly emphasized targeted delivery strategies that selectively functionalize specific cell populations while bypassing dysfunctional or degenerating cells. In the retina, MCO-010 is delivered by intravitreal injection to selectively target surviving ON-bipolar cells, thereby circumventing the requirement for functional photoreceptors. Similar principles have been explored in other disease models, where engineered delivery systems have demonstrated the ability to improve targeting specificity and therapeutic efficacy through cellular selectivity [35].

## 5. Limitations of the Current Study

While the *Abca4^-/-^* mouse model demonstrates slower photoreceptor degeneration with a late-onset phenotype more analogous to dry-AMD than to early-onset juvenile Stargardt disease, it remains a valuable tool for investigating the cellular and molecular consequences of *ABCA4* deficiency and testing therapeutic interventions in a controlled setting. Importantly, the functional improvements observed in this model should be interpreted within the context of its limitations—particularly the preservation of residual photoreceptor function—and the distinction must be maintained between the monogenic early-onset Stargardt disease caused by *ABCA4* mutations in humans and the late-onset dry-AMD with which *ABCA4* variants are associated. Nevertheless, insights gained from the *Abca4^-/-^* model have relevance to both conditions and may inform strategies applicable to human patients [36].

Another limitation of this study is the lack of a concurrent noninjected control cohort alongside the treated and AAV2-control groups, making it challenging to definitively interpret whether procedural differences or potential toxicity introduced by the injection itself influenced the study outcomes. However, to establish a baseline for disease progression, we included data demonstrating that noninjected *Abca4* mice show no change in ambient-light guided locomotion over time. Moreover, the AAV2-control group, which received the same dose, showed no injection-related damage in OCT imaging or acute ERG assessments, mitigating concerns about negative responses stemming from the injection procedure.

## 6. Conclusions

This study demonstrates that optogenetic activation of higher-order retina neurons by MCO-010 can enhance visual function in a Stargardt disease model, as evidenced by improved light sensitivity and visually guided behavior. Additionally, MCO-010-treated retinas maintained structural stability throughout the study period. These findings suggest potential therapeutic applications of optogenetic approaches for Stargardt disease. Optogenetic gene therapy-based treatment offers a unique approach to bypass dysfunctional or degenerated photoreceptors in retinal degenerative disorders. Since this approach focuses on disease phenotype instead of a specific genotypic deficit, it is applicable to a broad spectrum of retinal degenerations and can be a disease-agnostic treatment for other outer retinal degenerative diseases.

## Data Availability

The original contributions presented in the study are included in the article/Supplementary Material, further inquiries can be directed to the corresponding author.

## Supplementary Materials

The following supporting information can be downloaded at: https://www.mdpi.com/article/10.3390/bioengineering13060660/s1. Figure S1: Electroretinogram in *Abca4* mice, intravitreally injected with vehicle control or MCO-010. Raw profiles of scotopic ERG in response to white light stimulation at 0.01, 0.1, 3, 6, and 25 cd. s/m^2^ in (A) AAV2 vehicle control, and (B) MCO-010 treated Abca4 mice at 12 weeks after injection (age: 30 weeks old); Figure S2: *Abca4* mice in radial arm water maze before and after AAV2-Vehicle injection. Time-lapse pictures of ambient-light guided locomotion in radial water-maze setup: (A) baseline, and (B) 12 weeks after AAV2-vehicle injection (age: 30 weeks). Light intensity: Center (7 µW/mm^2^), Side, and Arm (5 µW/mm^2^). The mouse has been marked by an ellipse in each frame. * Cut-off time for each trial is 50 s; Figure S3: Noninjected *Abca4* mice in radial arm water maze. Change in latency of noninjected control *Abca4* mice to find lighted-platform in radial water-maze, with starting position at Center and Side arm of the maze. BL1-4: Baseline, separated by 1 week; Light intensity: Center (7 µW/mm^2^), Side, and Arm (5 µW/mm^2^). N = 5, Av ± SEM; Figure S4: *Abca4* mice in radial arm water maze before and after MCO-010 injection. Time-lapse pictures of ambient-light guided locomotion in radial water-maze setup: (A) baseline, and (B) 12 weeks after MCO-010 injection (age: 30 weeks). Light intensity: Center (7 µW/mm^2^), Side, and Arm (5 µW/mm^2^). The mouse has been marked by an ellipse in each frame; Figure S5: Longitudinally measured OCT Images *Abca4* mice before and after AAV2-control and MCO-010 injection. Top row: Representative B-Scan OCT images of control *Abca4* mice at Baseline (age: 18 weeks) and Weeks 8, 16, and 24 after injection of AAV2-control. Bottom row: Representative B-Scan OCT images of *Abca4* mice at Baseline (age: 18 weeks) and Weeks 8, 16, and 24 after 1.7 × 10^9^ gc/eye (1.14 × 10^12^ gc/mL, 1.5 µL) MCO-010 injection. Scale bar: 300 µm.
