# Supplementary material for: Therapeutic Efficacy of Multi-Characteristic Opsin Gene Therapy in a Mouse Model of Stargardt Disease
Source: Bioengineering (Basel). 2026 Jun 4;13(6):660. doi: 10.3390/bioengineering13060660 (PMC13296013; doi:10.3390/bioengineering13060660)
Supplement: Supplementary file 1 [file bioengineering-13-00660-s001.zip › bioengineering-4296739-supplementary.pdf]

## SUPPLEMENTARY INFORMATION

# Therapeutic Efficacy of Multi-characteristic opsin gene therapy in a mouse model of Stargardt disease

**Samarendra Mohanty [1,2]*, Subrata Batabyal [2], Sanghoon Kim [2], Michael Carlson [2] and Adnan Dibas [2]**

[1] Nanoscope Therapeutics Inc, 2777 N. Stemmons Fwy, Dallas, Texas, USA, 75207
[2] Nanoscope Technologies LLC, 1312 Brown Trail, Bedford, Texas, USA, 76022
* Correspondence: Author E-mail: smohanty@nanostherapeutics.com

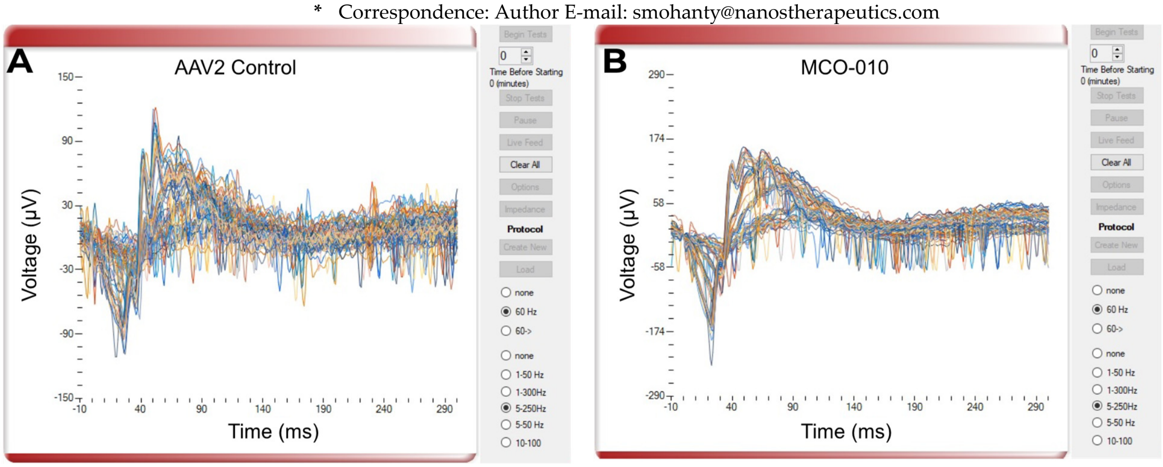


**Suppl. Fig. S1. Electroretinogram in *Abca4* mice, intravitreally injected with vehicle control or MCO-010.** Raw profiles of scotopic ERG in response to white light stimulation at 0.01, 0.1, 3, 6, and 25 cd. s/m$^2$ in (A) AAV2 vehicle control, and (B) MCO-010 treated *Abca4* mice at 12 weeks after injection (age: 30 weeks old).

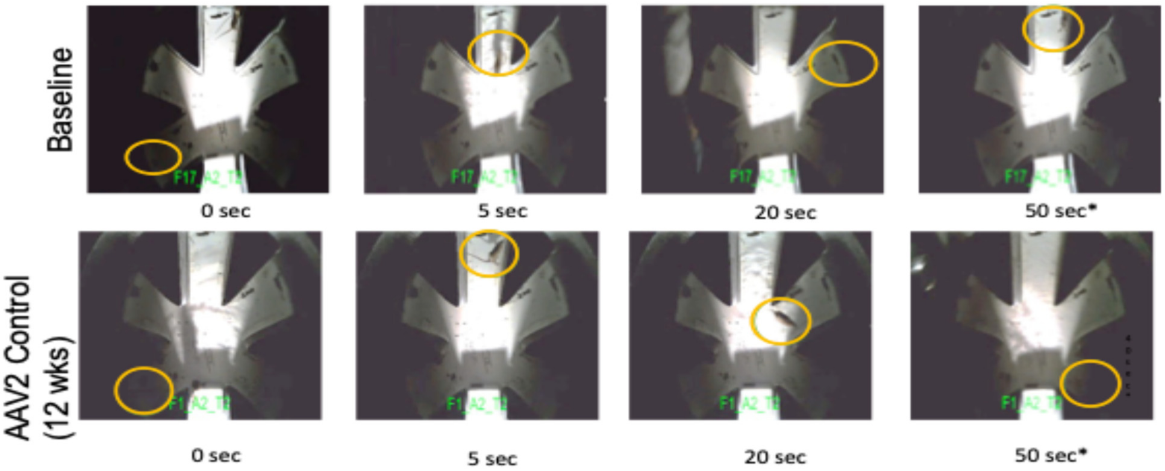


**Suppl. Fig. S2. *Abca4* mice in radial arm water maze before and after AAV2-Vehicle injection.** Time-lapse pictures of ambient-light guided locomotion in radial water-maze setup: (A) baseline, and (B) 12 weeks after AAV2-vehicle injection (age: 30 weeks). Light intensity: Center (7 μW/$mm^2$), Side, and Arm (5 μW/$mm^2$). The mouse has been marked by an ellipse in each frame. * Cut-off time for each trial is 50 sec.

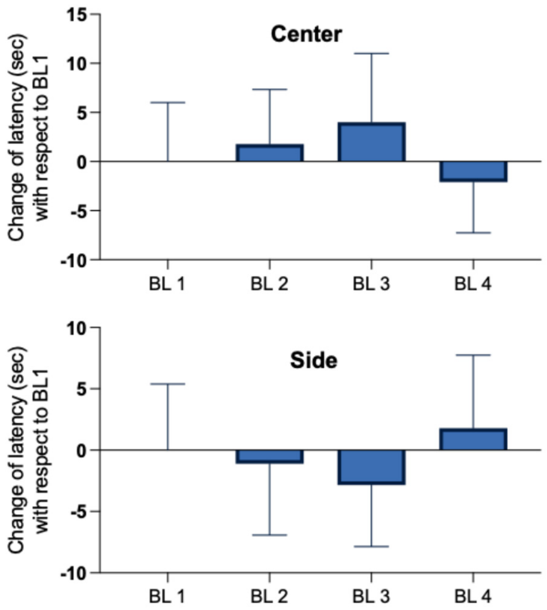


**Suppl. Fig. S3. Noninjected *Abca4* mice in radial arm water maze.** Change in latency of noninjected control *Abca4* mice to find lighted-platform in radial water-maze, with starting position at Center and Side arm of the maze. BL1-4: Baseline, separated by 1 week; Light intensity: Center (7 μW/mm$^2$), Side, and Arm (5 μW/mm$^2$). N=5, Av ± SEM.

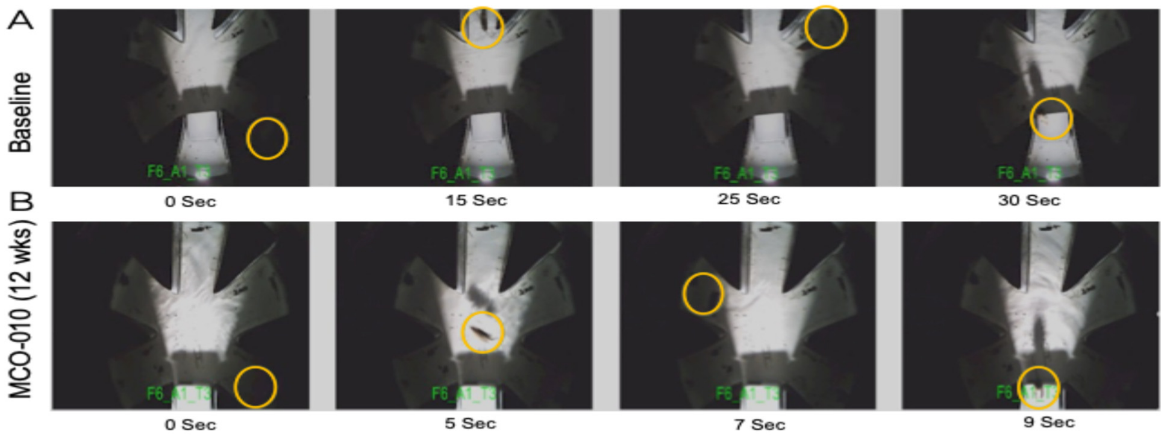


**Suppl. Fig. S4. *Abca4* mice in radial arm water maze before and after MCO-010 injection.** Time-lapse pictures of ambient-light guided locomotion in radial water-maze setup: (A) baseline, and (B) 12 weeks after MCO-010 injection (age: 30 weeks). Light intensity: Center (7 μW/mm$^2$), Side, and Arm (5 μW/mm$^2$). The mouse has been marked by an ellipse in each frame.

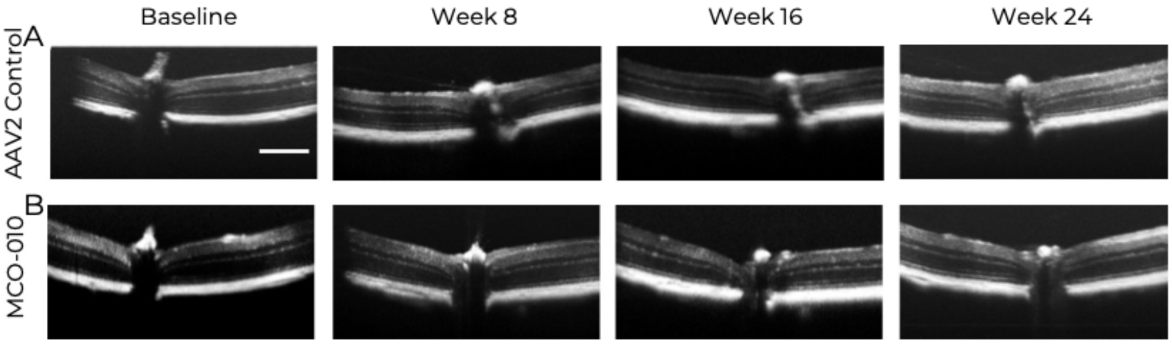


**Suppl. Fig. S5. Longitudinally measured OCT Images *Abca4* mice before and after AAV2-control and MCO-010 injection.** Top row: Representative B-Scan OCT images of control *Abca4* mice at Baseline (age: 18 weeks) and Weeks 8, 16, and 24 after injection of AAV2-control. Bottom row: Representative B-Scan OCT images of *Abca4* mice at Baseline (age: 18 weeks) and Weeks 8, 16, and 24 after 1.7E9 gc/eye (1.14E12 gc/ml, 1.5 µl) MCO-010 injection. Scale bar: 300 µm.
